# A comparative analysis of skin bacteria in healthy individuals through traditional cultivation and high-throughput sequencing techniques

**DOI:** 10.1038/s41598-026-51175-w

**Published:** 2026-04-30

**Authors:** Jing Feng, Shaolu Zhou, Jiancong Huang, Xia Wen, Gang Zhou, Guifang Zhang, Qingshan Shi, Xiaobao Xie

**Affiliations:** Guangdong Provincial Key Laboratory of Microbial Culture Collection and Application, State Key Laboratory of Applied Microbiology Southern China, Guangdong Detection Center of Microbiology, Institute of Microbiology, Guangdong Academy of Sciences, Guangzhou, 510070 China

**Keywords:** Skin microbiome, Skin health, Bacterial diversity, Microbial cultivation, Isolation and identification, High-throughput sequencing, Microbiology, Molecular biology

## Abstract

The human skin microbiota is intricately linked to the microenvironmental niche of its habitat. Different skin sites may harbor distinct bacterial community structures due to their unique microenvironments. To elucidate the site-specific distribution patterns of skin bacterial communities, this study employed an integrated approach combining traditional microbial cultivation methods with 16S rRNA gene high-throughput sequencing to analyze 105 skin swab samples collected from the forehead, volar forearm, and scalp of 35 healthy individuals. A total of 675 bacterial strains encompassing 41 species and 18 genera were isolated by traditional culture-based methods. *Staphylococcu*s and *Cutibacterium* were the dominant genera in different skin sites, with *S. capitis*, *S. epidermidis*, *S. hominis*, and *C. acnes* identified as the predominant species. High-throughput sequencing analyses revealed that the forehead and forearm harbored similar microbiota profiles, while the scalp displayed a more distinct bacterial community characterized by reduced alpha diversity and elevated relative abundances of *Cutibacterium*, *Staphylococcus*, and *Lawsonella* genera. The findings from both traditional cultivation methods and high-throughput sequencing suggested that the human skin bacterial community exhibits strong site-specificity. In contrast to the significant variations observed across different skin sites, gender and age exerted minimal effects on microbiota composition. These results suggest that microenvironmental factors play a more substantial role than age- and gender-related factors in shaping the diversity and community structure of the human skin microbiota.

**Supplementary Information:**

The online version contains supplementary material available at 10.1038/s41598-026-51175-w.

## Introduction

The human skin constitutes the body’s largest organ and functions as the principal defense mechanism against exogenous pathogens and harmful substances^1^. As the interface between the body and the external environment, the skin surface is home to various microorganisms like bacteria, fungi, and viruses^2^. Similar to the intestinal microbiota, the skin microbiota is crucial for protecting against pathogen invasion, modulating the immune system, and decomposing natural products. It is an integral part of the multi-dimensional barrier function of the skin^3^. The skin micro-ecological environment, which consists of various microorganisms, desquamated cells from the stratum corneum, and secretions from sweat and sebaceous glands, maintains the ecological balance between microbial communities and skin tissues, significantly influencing the immune and regulatory systems of the human skin^4^. Significant changes in internal and external environments can disrupt this balance, leading to micro-ecological imbalances that may trigger skin disorders such as atopic dermatitis, acne, psoriasis, and other infections^5–11^.

The composition of the human skin microbial community is influenced by a multitude of factors, including age, gender, genetic inheritance, immune response, and environmental conditions such as prolonged sun exposure and seawater contact, in addition to the microenvironment of particular skin sites^12–15^. Based on the physiological characteristics such as temperature, humidity, pH, sweat secretion, sebum production, and hair presence, the human skin microenvironment can be categorized into four main types: dry areas, moist areas, oily areas, and other specialized regions^2,12,16^. The scalp, characterized by its tendency to produce both sebum and sweat along with dense hair follicles, possesses a unique microenvironment that may harbor a distinct microbial community compared to other skin sites. Although research on the microbiome in dry, moist and oily skin areas has been relatively abundant, studies focusing on the microbial community diversity and population composition of the scalp remain limited. Further investigation into the bacterial types and compositions across different skin sites is crucial for understanding the relationship between skin health and disease. Moreover, a deeper insight into the bacterial characteristics of various skin areas can provide a scientific foundation for personalized skin care and treatment strategies.

Research methodologies for the human skin microbiome can be broadly categorized into two main types: traditional culture-based methods and modern genomic sequencing techniques, such as amplicon sequencing and shotgun metagenomics. High-throughput sequencing technology utilizing 16S/ITS rRNA gene amplification allows for rapid and efficient genetic analysis of all microorganisms present in a sample. This technology has reached a level of maturity that provides robust support for comprehensive investigations into the composition, relative abundance, and potential functions of the skin microbiome, establishing it as the predominant method in current skin microbiome research. However, the limitation of this technology is that the sequencing results are usually semi-quantitative and cannot distinguish between live and dead bacteria, and cannot obtain live cultured strains^16,17^.

Although traditional cultivation methods exhibit low efficiency and tend to seriously underestimate the total diversity of the community, they remain a crucial approach for obtaining various live cultured strains and are indispensable for researches on microbial communities. The acquisition of live strains serves as the foundation for subsequent specific analyses, including evolutionary diversity assessments, drug resistance evaluations, and investigations into synergistic or antagonistic interactions among different strains. These areas represent burgeoning frontiers in current research on the human skin microbiome^18,19^.

The integration of traditional culture-based methods and modern sequencing technologies, by leveraging their complementary strengths, effectively overcomes the limitations inherent in single technical approaches. This synergistic strategy not only enhances the comprehensiveness and accuracy of microbial community analysis but also deepens our understanding of the dynamic functional relationships between skin microbiota and the health-disease continuum. Building on this foundation, our study employed an integrated approach combining traditional cultivation methods with high-throughput 16S rRNA gene sequencing to systematically characterize bacterial community diversity and composition across three representative human skin sites (forehead, volar forearm, and scalp). The research primarily aimed to identify significant variations in bacterial diversity and predominant genera among these sites and to delineate the site-specific distribution patterns of skin bacterial communities. Additionally, we assessed the potential influence of gender and age on bacterial community composition within distinct skin microenvironments. Furthermore, we cultivated 675 viable bacterial isolates from these sites using conventional culture methods, establishing a valuable microbial resource for subsequent functional characterization and mechanistic investigations.

## Materials and methods

### Subject recruitment

Thirty-five healthy volunteers with intact skin were recruited, all of whom had been living in Guangzhou (Guangdong, China) for at least one year. Among these participants, 18 were female and 17 were male, aged 24 to 49. Specifically, there were 14 volunteers aged 24–29 years (20s), 11 volunteers aged 30–39 years (30s), and 10 volunteers aged 40–49 years (40s) (Table S1). The selection criteria for volunteers included the absence of any skin lesions such as wounds, erythema, acne, or psoriasis, no dandruff or hair loss on the scalp, and no use of topical or systemic steroids, antibiotics, or antifungal agents within the past two months.

### Sample collection

Three distinct sites on each participant’s skin surface were selected for sampling: the forehead, the left volar forearm, and the scalp. To preserve the natural state of the skin microbiota and improve ecological validity, site-specific washout periods were designed according to the participants’ daily hygiene practices. Accordingly, participants were instructed to avoid washing the forehead for 12 h prior to sampling (corresponding to the typical twice-daily facial cleansing habit), and to avoid washing both the forearm and scalp for 24 h (corresponding to the once-daily cleansing habit for these areas).

Sampling was performed following a standardized protocol using rayon-tipped swabs moistened with sterile saline. Each swab was rotated firmly with uniform pressure over a 4 cm × 4 cm skin area for 30 s. At each sampling site, paried specimens were collected: one for microbial isolation and cultivation, and the other for high-throughput sequencing. For the forehead and forearm, paired samples were obtained from adjacent positions within the same delineated area. For the scalp, the area was divided into four quadrants (anterior, posterior, left, and right) using a cross-shaped division method, with one anterior and one posterior quadrant randomly selected for sampling. Each sample was collected using a separately packaged sterile swab. Additionally, swabs moistened with sterile saline solution were used as negative controls.

Immediately after collection, the swab head was detached and placed into a dry, sterile 15 mL centrifuge tube (without any transport medium), following a one-sample-per-container storage protocol to maintain the sample independence. To minimize changes in microbial composition, all samples were processed within 10 min, with sequencing samples being snap-frozen at -80 °C and culture samples stored at 4 °C. A total of 210 samples (70 per site) were collected and evenly allocated for cultivation (*n* = 105; 35 per site) and sequencing (*n* = 105; 35 per site). All sample collection, strain isolation, identification, and DNA extraction were performed in Guangzhou, China, from June to August 2024 (Table S2).

The procedures followed were in accordance with the ethical standards of the responsible committees on human experimentation (institutional and national) and with the Helsinki Declaration of 1975, as revised in 2013. All participants provided informed consent before participating in the study, and the study was approved by the ethics committee of the Institute of Microbiology, Guangdong Academy of Sciences. The data collected were kept confidential and only used for research purposes.

### Preparation of medium

The tryptic soy agar (TSA, containing 15 g/L tryptone, 5 g/L soya peptone, 5 g/L NaCl, and 15 g/L agar; HuanKai Microbial, China), 5% sheep blood agar (TSA-SB, TSA supplemented with 5% defibrinated sheep blood; HuanKai Microbial, China), and anaerobic agar (AA, containing 20 g/L peptone, 5 g/L NaCl, 0.4 g/L L-cystine, 10 g/L glucose, 2 g/L sodium thioacetate, 1 g/L sodium bisulfite formaldehyde complex, 0.002 g/L methylene blue, and 20 g/L agar; Hopebio, China) were prepared according to standard methods and sterilized at 121 ℃, 15 psi for 20 min. After autoclaving, the media were poured into plates under aseptic conditions.

### Isolation and purification of bacterial strains

5 mL of sterile physiological saline were added to the centrifuge tube containing the skin swab samples, followed by thorough vortexing to ensure homogeneity. 10-fold and 100-fold serial dilutions were performed on the bacterial suspension. 100 µL of each diluted suspension was then withdrawn and spread evenly onto TSA, TSA-SB, and AA plates. The TSA and TSA-SB plates were incubated at 37 °C for 24 to 48 h in a SHP-250 biochemical incubator (HuanKai Microbial, China), while the AA plates were placed in an A35 anaerobic incubator (Don Whitley Scientific, Bingley, UK) at the same temperature for 7 to 14 days. After incubation, single colonies were removed from these plates and sub-cultured by streaking on the surface of TSA and AA plates for purification and preservation. For long-term preservation and to ensure genetic integrity, bacterial suspensions of each strain were mixed with sterile glycerol to achieve a final concentration of 20% (v/v). The mixture was gently inverted for homogeneous distribution and equilibrated at 4 °C for 10 min. Aliquots (600 µL) were aseptically transferred into pre-labeled cryovials (2.0 mL) and flash-frozen in liquid nitrogen prior to storage at -80 °C.

### Identification of bacterial strains

Purified strains were reactivated and streaked onto TSA or AA plates, and then single colonies were selected and genomic DNA was rapidly extracted using the AmPure Microbial DNA Kit (Magen Biotech, China). The 16S rRNA genes were subjected to polymerase chain reaction amplification using the universal primers 27F (5’-AGAGTTTGATCCTGGCTCAG-3’) and 1492R (5’-GGTTACCTTGTTACGACTT-3’)^20^. Thirty PCR cycles were conducted using the 2×Taq PCR Mastermix (Vazyme Biotech, China), with each cycle consisting of three steps: 95 ℃ for 15 s, 53 ℃ for 15 s, and 72 ℃ for 1.5 min. PCR products were purified and sequenced using Sanger sequencing on an ABI 3730XL DNA Analyzer (Applied Biosystems, Foster City, CA, USA) by Tsingke Biological Technology (Beijing, China). The obtained sequences were compared with available 16S rRNA gene sequences in NCBI (http://www.ncbi.nlm.nih.gov/) and EzBioCloud (https://www.ezbiocloud.net/) databases using the Basic Local Alignment Search Tool (BLAST) programs to determine an approximate phylogenetic affiliation of each strain^21^. When the top three matching BLAST hits were from the same species and were > 99% similar to the query sequence, this species name was assigned to the selected strain. DNA sequences of 675 strains generated in this study have been deposited in GenBank under accession numbers PV224589 to PV225263.

### DNA extraction and PCR amplification for high-throughput sequencing

Total microbial DNA from skin swab samples was extracted using Magnetic Viral Genomic DNA Kit (Tiangen Biotech, China) following the manufacturer’s instructions. The PCR amplification was performed for the 16S rRNA genes V3 + V4 variable region using the universal primers 338 F (5’-ACTCCTACGGGAGGCAGCA-3’) and 806R (5’-GGACTACHVGGGTWTCTAAT-3’)^22^. PCR conditions were: 95 ℃ for 5 min, followed by 25 cycles of 95 ℃ for 30 s, 50 ℃ for 30 s, 72 ℃ for 40 s, and a final elongation step at 72 ℃ for 7 min. The success of the PCR products was verified with 1.8% agarose gel electrophoresis, and then recovered and purified using the Monarch^®^ DNA Gel Extraction Kit (New England Biolabs, USA). High-throughput sequencing analysis of bacterial 16S rRNA genes was performed on the Illumina NovaSeq6000 platform at Biomarker Technologies Corporation (Beijing, China).

### Amplicon data and statistical analysis

Microbiome bioinformatics analyses were conducted using QIIME2 (version 2020.6) and R package (version 2.15.3)^23^. Raw sequence data were quality filtered and primer sequences were removed using the Trimmomatic (version 0.33) and Cutadapt (version 1.9.1) plugins, respectively^24,25^. The processed sequences were then denoised, merged, and chimeras identified and removed to obtain amplicon sequence variants (ASVs) using the DADA2 plugin^26^. ASVs with a total frequency of fewer than 2 reads were filtered out to minimize potential sequencing artifacts. Taxonomy was assigned to ASVs using the classify-sklearn naïve Bayes taxonomy classifier in feature-classifier plugin against the SILVA Database (Release 138)^27,28^. A Venn diagram was generated using the VennDiagram R package to illustrate the shared and unique ASVs across different groups. To ensure comparability, all samples were rarefied to the same sequencing depth (10,000 reads per sample) for further diversity analysis. Alpha diversity (Shannon index, Simpson index, and Observed species) were assessed and visualized as violin plots using the rarefied data counts in QIIME2^29^. Wilcoxon rank-sum tests were employed to assess statistical differences between groups. Principal coordinate analysis (PCoA) for beta diversity based on weighted UniFrac distance was constructed and visualized using the R package. Significant differences in bacterial composition between groups in the PCoA were assessed by permutational multivariate analysis of variance (PERMANOVA) using the adonis function with 9999 permutations^30^. Prior to PERMANOVA, homogeneity of multivariate dispersions was verified through PERMDISP analysis (9999 permutations)^31^. The relative abundance (%) of each taxon was calculated by comparing the number of sequences assigned to that taxon with the total number of sequences of the sample. Differential abundance at the phylum and genus levels was analyzed using analysis of variance (ANOVA) with Benjamini-Hochberg FDR (BH-FDR) correction for multiple comparisons. All aforementioned data analyses were conducted on the BMKCloud platform (www.biocloud.net). The raw high-throughput amplicon sequencing data have been deposited in the NCBI Sequence Read Archive (SRA) under the accession number PRJNA1252278.

## Results

### Classification and identification of bacterial isolates across diverse skin sites

Using culture-based techniques, we performed a comprehensive analysis of bacterial populations from 105 skin swab samples. The numerical distribution and taxonomic classification of bacterial strains isolated from distinct skin regions, along with species-level percentages, were summarized in Table 1. The percentage of each species was computed as the ratio of its strain count to the total number of isolates obtained from 35 samples per sampling site. It is important to note that these percentages only represent culture-based recovery rates, which may favor fast-growing aerobic species and therefore might not accurately reflect the true relative abundances within the sampled communities.

A total of 675 bacterial strains encompassing 41 species and 18 genera were isolated and characterized from three skin sites. *Staphylococcus* (75.4% in forehead; 61.9% in forearm; 80.9% in scalp) and *Cutibacterium* (9.4% in forehead; 21.3% in forearm; 13.9% in scalp) were the predominant genera, collectively accounting for over 80% of the total culturable bacterial communities in each sampling site. The remaining 16 genera, including *Bacillus*, *Micrococcus*, and *Pseudomonas*, each constituted less than 5% of the total isolated strains. We identified 12 *Staphylococcus* species, with *S. epidermidis* and *S. capitis* being the highest abundant. The genus *Cutibacterium* comprised four species, dominated by *C. acnes*, which represented 78.9% of total genus abundance.

Notable differences in culturable bacterial community composition were observed across the three skin sites. The scalp yielded 408 bacterial strains (11 genera, 21 species) which was substantially higher than the 159 strains (14 genera, 24 species) recovered from the forehead and the 108 strains (10 genera, 20 species) from the forearm. The distribution of staphylococcal species revealed microenvironment-specific colonization patterns. To define dominant colonizers, we applied a 5% abundance threshold as an operational definition. This value lies within the center of the commonly used range (1%–10%) in microbial ecology for defining core or abundant populations. This threshold was set to simplify the presentation of dominant patterns and define primary staphylococcal species. Accordingly, the primary staphylococcal species in the forehead were *S. epidermidis* (37.1%), *S. capitis* (25.8%), and *S. warneri* (6.9%). The forearm bacteria exhibited a distinct composition, with *S. capitis* (22.2%), *S. hominis* (18.5%), *S. epidermidis* (11.1%), and *S. warneri* (7.4%) as the primary species. On the scalp, *S. capitis* was overwhelmingly predominant at 51.2%, followed by *S. epidermidis* (20.3%) and *S. caprae* (6.1%). These findings confirmed marked topographic variation in the culturable bacterial communities. In terms of taxonomic richness, the forehead supported the most diverse community, followed by the scalp, with the forearm being the least diverse. In contrast, the scalp yielded the highest number of bacterial isolates, indicating the highest bacterial density or culturable biomass among the three sites.

**Table 1:** Characterization of bacterial strains: taxonomic classification, quantitative distribution, and species-level percentages across skin sites.

Taxonomic classification		Numbers of strains in different site			Species-level percentages (%)		
Genus	Species	Forehead	Forearm	Scalp	Forehead	Forearm	Scalp
*Staphylococcus*	*Staphylococcus epidermidis*	59	12	83	37.1	11.1	20.3
	*Staphylococcus capitis*	41	24	209	25.8	22.2	51.2
	*Staphylococcus hominis*	1	20	1	0.6	18.5	0.2
	*Staphylococcus warneri*	11	8	7	6.9	7.4	1.7
	*Staphylococcus aureus*	4	0	0	2.5	0	0
	*Staphylococcus haemolyticus*	0	1	2	0	0.9	0.5
	*Staphylococcus lugdunensis*	1	0	0	0.6	0	0
	*Staphylococcus shinii*	0	1	0	0	0.9	0
	*Staphylococcus edaphicus*	3	0	0	1.9	0	0
	*Staphylococcus pasteuri*	0	0	1	0	0	0.2
	*Staphylococcus caprae*	0	1	25	0	0.9	6.1
	*Staphylococcus simulans*	0	0	3	0	0	0.7
*Cutibacterium*	*Cutibacterium acnes*	15	22	38	9.4	20.4	9.3
	*Cutibacterium granulosum*	0	1	4	0	0.9	1
	*Cutibacterium avidum*	0	0	1	0	0	0.2
	*Cutibacterium namnetense*	0	0	14	0	0	3.4
*Bacillus*	*Bacillus altitudini*s	2	3	0	1.3	2.8	0
	*Bacillus cereus*	1	1	0	0.6	0.9	0
	*Bacillus siamensis*	0	1	0	0	0.9	0
	*Bacillus tequilensis*	0	0	1	0	0	0.2
*Brevibacterium*	*Brevibacterium casei*	1	0	0	0.6	0	0
	*Brevibacterium epidermidis*	0	1	0	0	0.9	0
*Barrientosiimonas*	*Barrientosiimonas humi*	0	0	2	0	0	0.5
*Clostridium*	*Clostridium sulfidigenes*	0	0	5	0	0	1.2
*Dermacoccus*	*Dermacoccus barathri*	1	0	0	0.6	0	0
	*Dermacoccus nishinomiyaensis*	0	1	0	0	0.9	0
*Gordonia*	*Gordonia terrae*	1	0	0	0.6	0	0
*Janibacter*	*Janibacter indicus*	1	0	0	0.6	0	0
*Klebsiella*	*Klebsiella pneumoniae*	0	0	2	0	0	0.5
*Kocuria*	*Kocuria indica*	1	0	1	0.6	0	0.2
	*Kocuria palustris*	3	0	0	1.9	0	0
*Microbacterium*	*Microbacterium paraoxydans*	0	1	0	0	0.9	0
*Micrococcus*	*Micrococcus luteus*	1	4	1	0.6	3.7	0.2
*Pseudomonas*	*Pseudomonas aeruginosa*	2	1	0	1.3	0.9	0
	*Pseudomonas oryzihabitans*	0	0	4	0	0	1
*Raoultella*	*Raoultella terrigena*	1	0	0	0.6	0	0
*Rhodococcus*	*Rhodococcus corynebacterioides*	1	0	0	0.6	0	0
	*Rhodococcus kroppenstedtii*	1	0	0	0.6	0	0
*Roseomonas*	*Roseomonas mucosa*	4	1	3	2.5	0.9	0.7
*Rothia*	*Rothia amarae*	2	3	1	1.3	2.8	0.2
	*Rothia terrae*	1	1	0	0.6	0.9	0
	Total	159	108	408			

### Variations of bacterial communities across distinct skin sites based on high-throughput sequencing

**Fig. 1:**
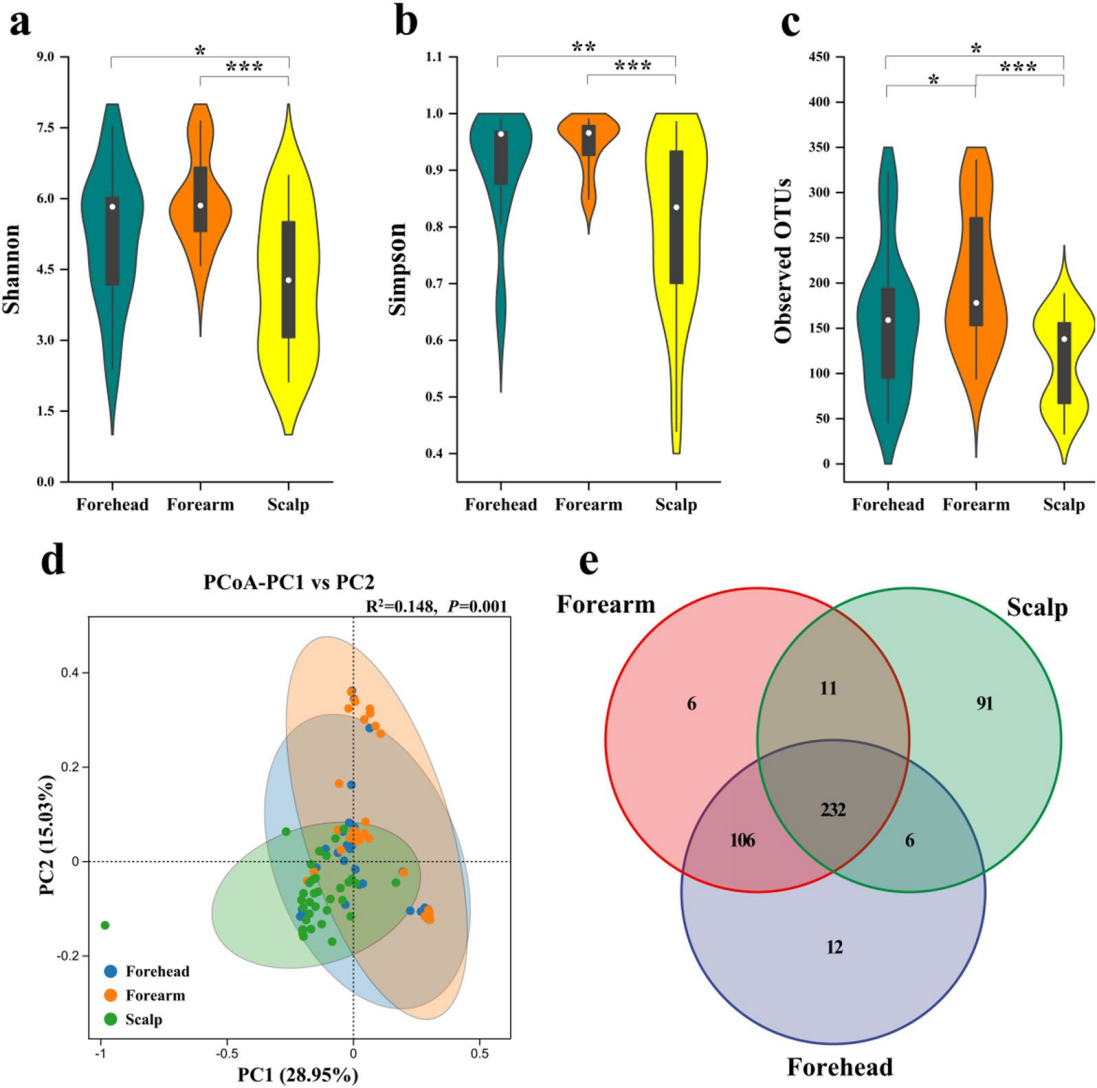
Diversity analysis of bacterial communities based on ASVs counts from the skin sites of the forehead, forearm, and scalp. (**a**) Shannon index of different skin sites, *P* values are calculated by two-sided Wilcoxon rank-sum tests; (**b**) Simpson index of different skin sites, *P* values are calculated by two-sided Wilcoxon rank-sum tests; (**c**) Observed ASVs index of different skin sites, *P* values are calculated by two-sided Wilcoxon rank-sum tests; (**d**) PCoA analysis of different skin sites, with statistical differences among groups performed using PERMANOVA; (**e**) Venn diagram displays the shared and unique genera in different skin sites. Significant differences are indicated as follows: *P < 0.05, **P < 0.01, and ***P < 0.001. Only statistically significant values (*P* < 0.05) are shown in the figures; non-significant results (*P* ≥ 0.05) are not represented.

After denoising using DADA2, an average of 69,946 high-quality 16S rRNA gene sequences per sample was obtained (range: 39,040–81,020). A total of 1,488 ASVs were obtained from 105 samples collected from the forehead, forearm, and scalp. Alpha diversity, beta diversity, and Venn diagram of bacterial genera were used to assess the differences in bacterial diversity among various skin sites (Fig. 1).

Alpha diversity analysis revealed significant variations in bacterial community diversity across three sites. The forearm demonstrated the highest Shannon index (5.96), followed by the forehead (5.19), while the scalp demonstrated the lowest diversity (4.22) (Fig. 1a). Simpson index analysis corroborated this trend, showing greater diversity in the forehead (0.90) and forearm (0.95) compared to the scalp (0.81; *P* < 0.01) (Fig. 1b). Observed ASVs quantification paralleled these findings, with the forearm (202) and forehead (157) containing significantly more ASVs than the scalp (116; *P* < 0.05) (Fig. 1c).

Beta diversity analysis based on weighted UniFrac distance revealed convergent clustering patterns between the forehead and forearm microbial communities, whereas scalp samples formed a distinct cluster. PCoA (PERMANOVA R²=0.148, *P* = 0.001) demonstrated significant overlap between the forehead and forearm communities, while the scalp community exhibited clear separation from other sites (Fig. 1d). Following confirmation of homogeneity in intergroup dispersion through PERMDISP (*P* = 0.821), PERMANOVA confirmed statistically significant differences in community structure among skin sites (*P* = 0.001). These differences were attributable to substantive compositional divergence rather than within-group dispersion variability.

Venn analysis of 464 bacterial genera showed that 232 (50.0%) core genera were shared across all sites. Pairwise comparisons revealed 338 (72.8%) shared genera between the forehead and forearm, compared to 238 (51.3%) and 243 (52.4%) in forehead-scalp and forearm-scalp pairs, respectively. Notably, the scalp exhibited 91 (19.6%) unique genera, a number significantly higher than the 12 (2.6%) unique genera identified on the forehead and 6 (1.3%) unique genera observed on the forearm (Fig. 1e). These findings revealed more conserved bacterial community structures between the forehead and forearm, whereas the scalp exhibited a distinct bacterial composition characterized by both reduced diversity and unique taxonomic features.

### Analysis of the compositional variations in bacterial communities at the phylum level across distinct skin sites

**Fig. 2:**
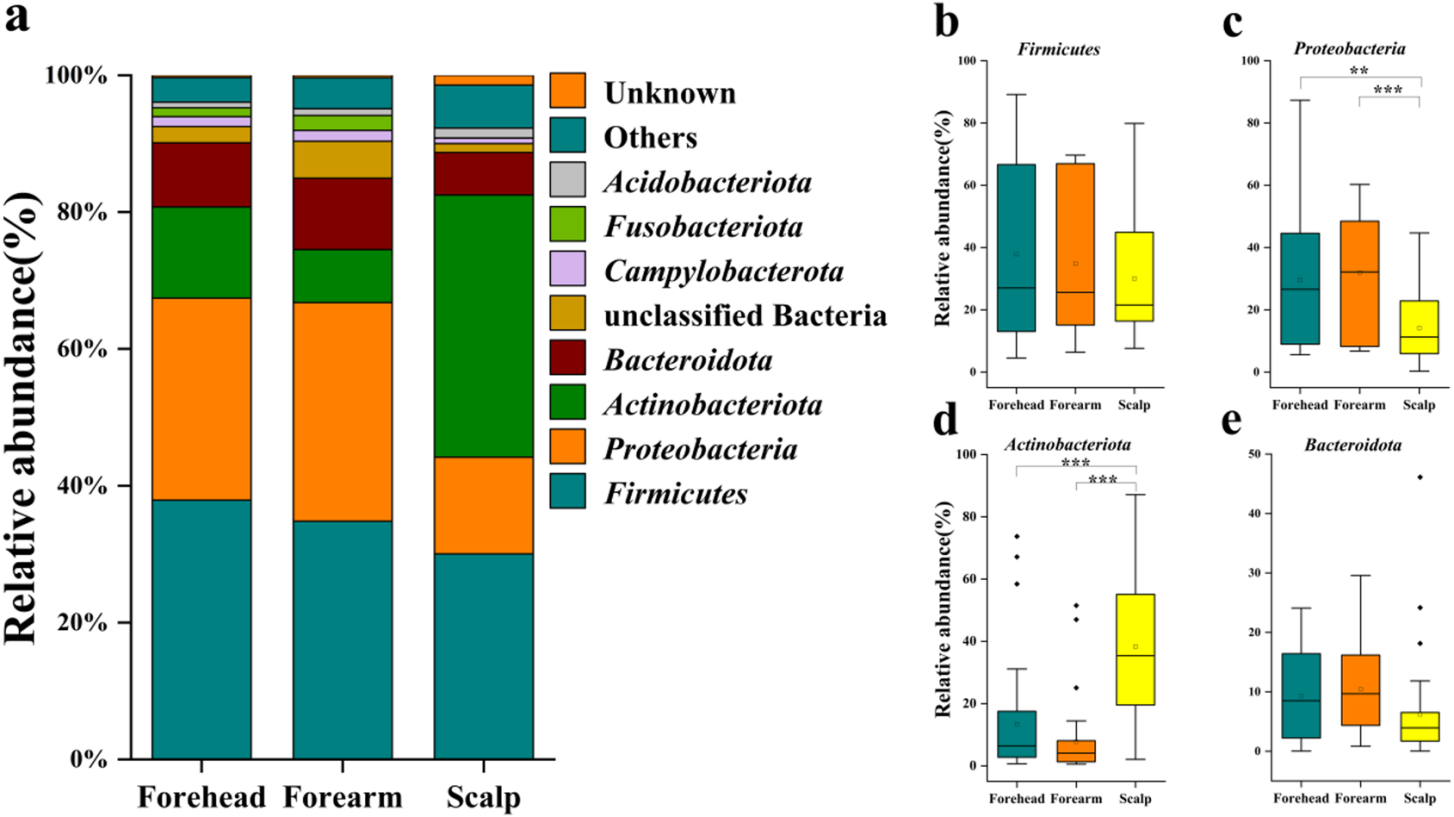
Differential bacterial composition at the phylum level across the forehead, forearm, and scalp sites. (**a**) Overall community profile showing phyla with relative abundances > 1.0%; the remaining phyla are grouped as “others”). (**b**–**e**) Statistical comparison of the four most abundant phyla: (**b**) *Firmicutes*, (**c**) *Proteobacteria*, (**d**) *Actinobacteriota*, (**e**) *Bacteroidota*. Statistical significance between groups was assessed using ANOVA with BH-FDR correction for multiple comparisons. Significant differences are indicated as follows: *P < 0.05, **P < 0.01, and ***P < 0.001. Only statistically significant values (*P* < 0.05) are shown in the figures, non-significant results (*P* ≥ 0.05) are not represented.

To investigate the similarities and differences in bacterial community structures across distinct human skin sites, we analyzed the bacterial composition at the phylum level in the forehead, forearm, and scalp. Sequences that were classified within the bacterial domain but could not be resolved to any specific taxonomic group were designated as “unclassified Bacteria”. In contrast, sequences showing insufficient similarity to any reference sequences in the database were categorized as “Unknown”, indicating that these sequences could not be classified using the currently available taxonomical reference data. Microorganisms with a relative abundance lower than 1.0% were grouped collectively as “Others” (Fig. 2a). *Firmicutes*, *Proteobacteria*, *Actinobacteriota*, and *Bacteroidota* were identified as the core dominant phyla shared among these three sites. These four phyla collectively accounted for 90.1%, 85.0%, and 88.7% of the total bacterial populations in the forehead, forearm, and scalp, respectively.

The statistical analysis of inter-group differences in the relative abundances of *Firmicutes*, *Proteobacteria*, *Actinobacteriota* and *Bacteroidota* revealed no significant differences between the forehead and forearm, indicating that these two sites exhibited similar microbial community structures (Fig. 2b–e). In comparison to the forehead and forearm, the relative abundances of *Firmicutes* and *Bacteroidota* were slightly lower on the scalp, but no significant differences were observed among these groups. Conversely, the relative abundance of *Proteobacteria* was significantly reduced compared to that in both the forehead and forearm. There was a notable increase in *Actinobacteriota* abundance on the scalp, with extremely significant differences observed when comparing to both other sites. The results from this differential analysis at high-abundance phylum levels further underscored that human skin microbiomes exhibit strong site-dependent characteristics.

### Variation in bacterial composition at the genus level is primarily driven by distinct skin sites

**Fig. 3:**
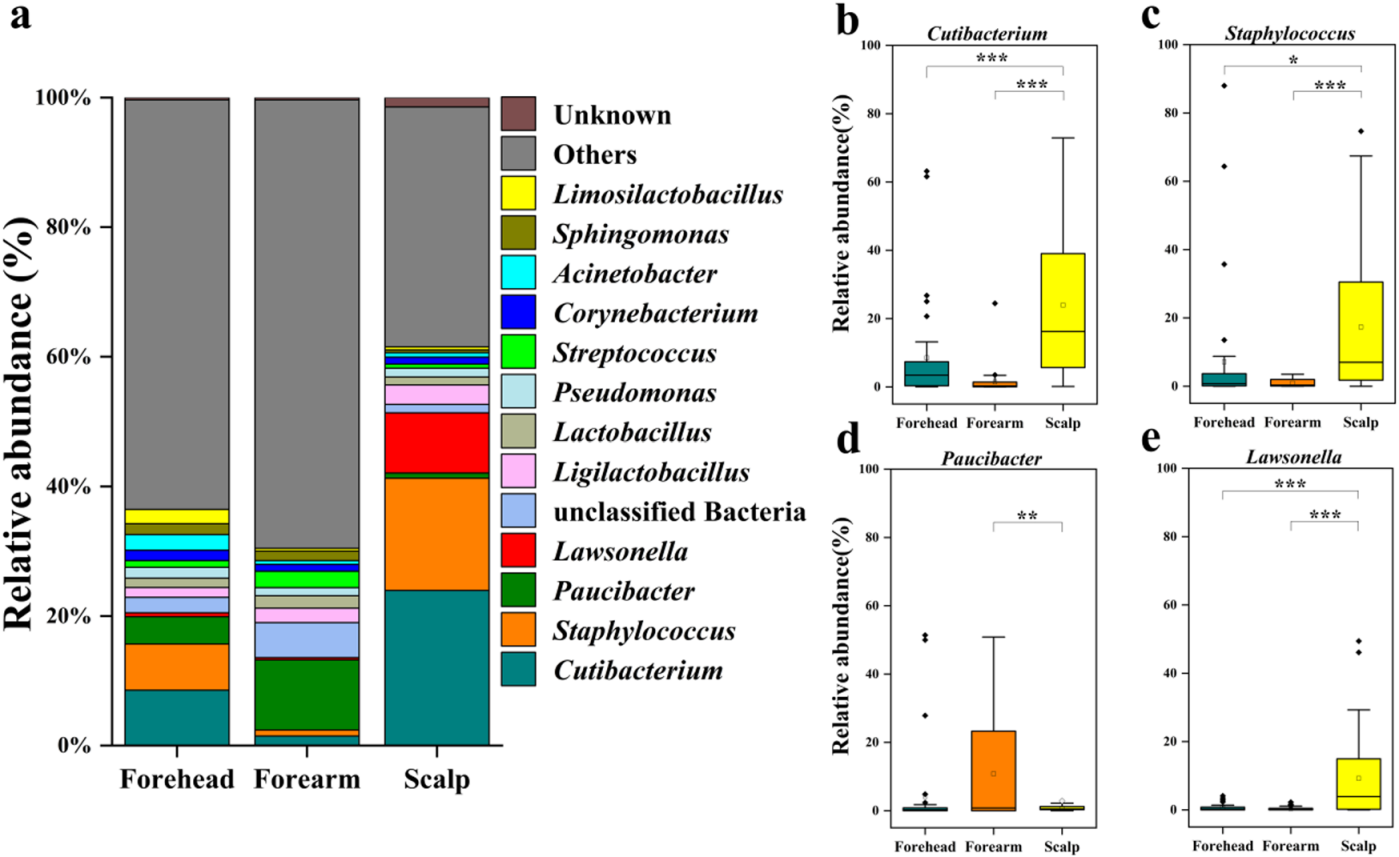
Composition and comparative analysis of bacterial communities at the genus level in the forehead, forearm, and scalp. (**a**) Bacterial community composition at the genus level. Genera with relative abundances exceeding 1.0% are displayed, while the remaining taxa are grouped as “Others. (**b**–**e**) Relative abundances and statistical comparisons of dominant genera: (**b**) *Cutibacterium*, (**c**) *Staphylococcus*, (**d**) *Paucibacter*, (**e**) *Lawsonella*. Statistical significance between groups was assessed using ANOVA with BH-FDR correction for multiple comparisons. Significant differences are indicated as follows: *P < 0.05, **P < 0.01, and ***P < 0.001. Only statistically significant values (*P* < 0.05) are shown in the figures, non-significant results (*P* ≥ 0.05) are not represented.

Notable differences in bacterial composition at the genus level were observed among different human skin sites (Fig. 3a). For clarity, only genera with a relative abundance exceeding 1.0% were plotted, with remaining taxa grouped and represented as “Others”. On the forehead, the three most abundant genera were *Cutibacterium* (8.5%), *Staphylococcus* (7.1%), and *Paucibacter* (4.2%), followed by *Acinetobacter*, *Limosilactobacillus*, and unclassified Bacteria (2.2%–2.4%). The forearm was dominated by *Paucibacter* (10.8%) and unclassified Bacteria (5.4%), with *Streptococcus* (2.5%) *and Ligilactobacillus* (2.2%) exhibiting moderate abundances. On the scalp, *Cutibacterium* was predominant (24.0%), followed by *Staphylococcus* (17.3%) and *Lawsonella* (9.2%); *Ligilactobacillus* accounted for 3.0%.

Phylogenetic analysis showed that among the genera with relative abundance > 1.0%, *Paucibacter*, *Pseudomonas*, *Acinetobacter*, and *Sphingomonas* all belonged to the phylum *Proteobacteria*. These genera were more abundant on the forehead and forearm than on the scalp, explaining the significantly lower relative abundance of *Proteobacteria* on the scalp. In contrast, *Cutibacterium* and *Lawsonella*, which belonged to the phylum *Actinobacteriota*, were substantially more abundant on the scalp, leading to a higher prevalence of *Actinobacteriota* there.

Further statistical analysis on the relative abundance of four predominant genera (*Cutibacterium*, *Staphylococcus*, *Paucibacter*, and *Lawsonella*) revealed no significant differences between the forehead and forearm (Fig. 3b–e). However, significant differences were observed between the scalp and both other sites. An exception was observed for *Paucibacter*, which showed no significant difference between the scalp and forehead. Specifically, the scalp had significantly higher abundances of *Cutibacterium*, *Staphylococcus*, and *Lawsonella* than the forehead and forearm; conversely, the relative abundance of *Paucibacter* was significantly lower on the scalp than on the forearm.

### Gender-related variations in bacterial community composition across distinct skin sites

**Fig. 4:**
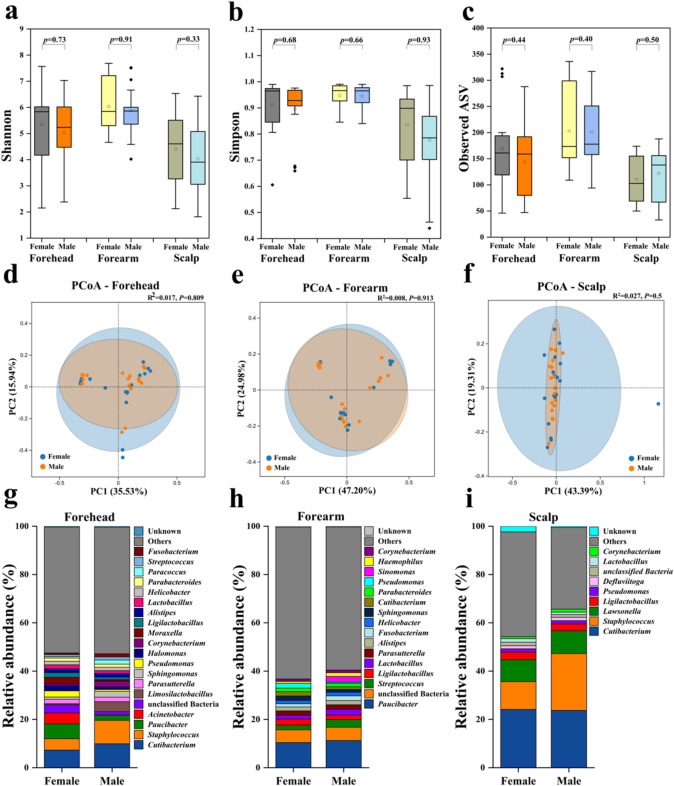
Analysis of bacterial community diversity and composition in healthy males and females across various skin sites. (**a**–**c**) Alpha diversity indices (Shannon, Simpson, Observed ASVs) with *P* values are calculated by two-sided Wilcoxon rank-sum tests; (**d**–**f**) Weighted UniFrac-based PCoA with PERMANOVA results; (**g**–**i**) Genus-level profiles (> 1.0% relative abundance) in forehead (**g**), forearm (**h**), and scalp (**i**).

To investigate gender-related variations in bacterial community diversity across distinct skin sites, we analyzed alpha diversity, beta diversity, and genus-level community structure in male (*n* = 17) and female (*n* = 18) volunteers at three sampling sites (Fig. 4).

Although females exhibit higher Shannon and Simpson indices than males, particularly on the forehead and scalp, these differences did not reach statistical significance (*P* > 0.05 for all alpha-diversity metrics; Fig. 4a–c). Beta diversity analysis, visualized using PCoA based on weighted UniFrac distances, revealed highly overlapping clusters between male and female groups across all sites. This was confirmed by PERMANOVA, which showed no significant gender-related differences in community composition (*P* > 0.05; Fig. 4d–f).

Genus-level analysis of bacterial communities across three skin sites revealed gender-associated distribution patterns, although none were statistically significant. On the forehead, predominant genera in males were *Cutibacterium* (9.88%), *Staphylococcus* (9.72%), and *Limosilactobacillus* (4.11%), while females exhibited a distinct profile dominated by *Cutibacterium* (7.29%), *Paucibacter* (6.09%), *Staphylococcus* (4.72%), and *Acinetobacter* (4.60%) (Fig. 4g). Despite notable numerical reductions in the relative abundances of *Staphylococcus* (51.45% lower) and *Cutibacterium* (26.24% lower) in females compared to males, ANOVA with BH-FDR correction showed no significant differences between groups (*P* > 0.05). In contrast, the forearm exhibited remarkable compositional similarity between genders, with both sharing the core genera *Paucibacter*, unclassified Bacteria, *Streptococcus*, and *Ligilactobacillus* (Fig. 4h). For each of these core genera, no statistically significant abundance differences were observed between genders. Similarly, scalp microbiota analysis indicated that both genders shared the core genera *Staphylococcus*, *Cutibacterium*, and *Lawsonella* (Fig. 4i). Although females showed a 51.17% reduction in the abundance of *Staphylococcus* compared to males, this difference was also not statistically significant (*P* > 0.05, ANOVA with BH-FDR correction).

### Age-related differences in bacterial community composition across distinct skin sites

**Fig. 5:**
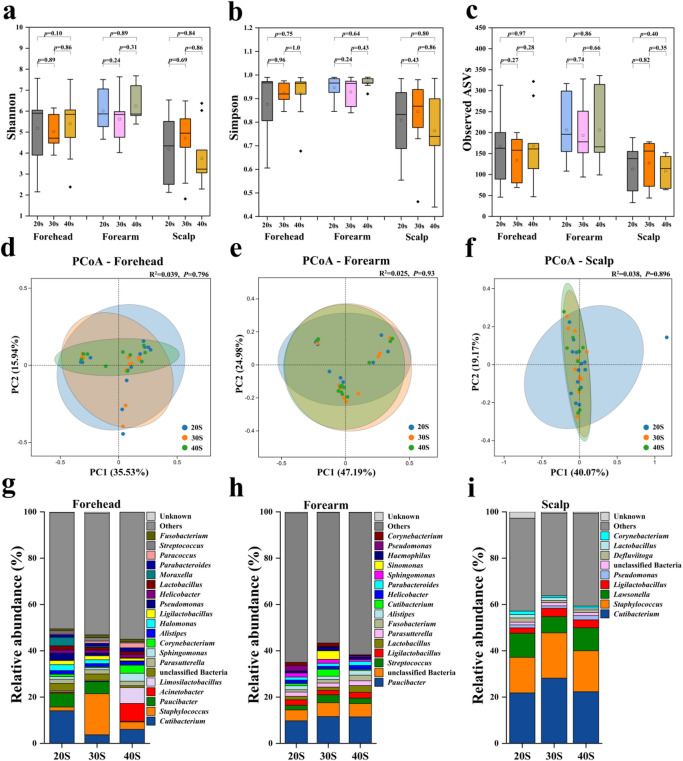
Analysis of bacterial community diversity and composition across age-stratified groups (20s, 30s, 40s) in distinct skin sites. (**a**–**c**) Alpha diversity indices (Shannon, Simpson, Observed ASVs) with *P* values are calculated by two-sided Wilcoxon rank-sum tests; (**d**–**f**) Weighted UniFrac-based PCoA with PERMANOVA results; (**g**–**i**) Genus-level profiles (> 1.0% relative abundance) in forehead (**g**), forearm (**h**), and scalp (**i**).

To examine age-related effects on skin bacterial diversity, participants from forehead, forearm, and scalp sites were categorized into three age groups: 20s group (24–29 years, *n* = 14), 30s group (31–39 years, *n* = 11), and 40s group (40–49 years, *n* = 10). Comparative analyses of alpha diversity, beta diversity, and genus-level community structure among these age groups are presented in Fig. 5.

Alpha diversity analysis revealed no significant differences in Shannon index, Simpson index, or ASV counts across age groups at any skin sites (*P* > 0.05; Fig. 5a–c). Weighted UniFrac-based PCoA demonstrated overlapping clustering patterns among age groups within each site, supported by PERMANOVA results showing non-significant differences (*P* > 0.05) in bacterial communities across forehead, forearm, and scalp sites among the three age groups (Fig. 5d–f).

Genus-level compositional analysis revealed distinct distribution patterns across age groups, although none were statistically significant. At the forehead, the 20s group was dominated by *Cutibacterium*, *Paucibacter*, and unclassified Bacteria. The 30s group was primarily dominated by *Staphylococcus*, *Paucibacter*, and *Cutibacterium*, while the 40s group showed dominance of *Acinetobacter*, *Limosilactobacillus*, *Cutibacterium*, and *Staphylococcus* (Fig. 5g). Notably, the mean abundance of *Staphylococcus* was highest in the 30s group (17.72%), which was approximately 11.1 times and 5.6 times that of the 20s (1.60%) and 40s (3.19%) groups, respectively. However, these differences were not statistically significant, likely due to exceptionally high intra-group variability in the 30s group (SD = 29.74%). Concurrently, the mean abundance of *Cutibacterium* in the 30s group (3.71%) was 73.6% and 38.8% lower than that in the 20s (14.09%) and 40s (6.07%) groups, respectively, with neither change reaching statistical significance (*P* > 0.05). The forearm bacterial community composition remained highly conserved across all age groups, consistently dominated by *Paucibacter*, unclassified Bacteria, and *Streptococcus*. Cross-age variations in the relative abundances of these genera were minimal (all < 2.0%), further indicating a high degree of site-specific stability (Fig. 5h). At the scalp site, all age groups shared *Cutibacterium*, *Staphylococcus*, and *Lawsonella* as core dominant genera (Fig. 5i). Specifically, compared to the 30s and 40s groups, the mean abundance of *Cutibacterium* in the 20s group was 22.6% and 2.2% lower, respectively. Similarly, the mean *Staphylococcus* abundance in the 20s group was 21.8% and 13.4% lower. None of these differences were statistically significant (*P* > 0.05).

## Discussion

In this study, we combined traditional culture-based methods with high-throughput sequencing techniques to analyze variations in bacterial community diversity across the forehead, forearm, and scalp. We further evaluated the site-specific distribution patterns of skin bacterial communities and examined the influence of gender and age on bacterial community composition within distinct skin sites.

Although traditional culture-based methods are less frequently utilized in human skin microbiome research due to their inherent limitations in efficiency and potential underestimation of microbial diversity, our study successfully isolated 675 bacterial strains using this approach. This achievement provides a valuable resource for subsequent functional and mechanistic investigations. Notably, the culture-based analysis revealed significant intersite variations, particularly in the microenvironment-specific distributions of staphylococcal species. Although *Staphylococcus* and *Cutibacterium* were identified as core genera across all sampled sites, *S. capitis* exhibited scalp-specific predominance, indicating distinct bacterial community characteristics in scalp site compared with forehead and forearm areas.

Substantial discrepancies were observed between culture-based and sequencing-based assessments of bacterial diversity. Cultivation methods revealed that the scalp yielded the highest number of strains (408 strains), suggesting the highest cultivable biomass, while the forearm yielded the fewest isolates (108 strains), indicating the least diverse cultivable community structure among the three sites. In contrast, high-throughput sequencing analysis demonstrated a complete reversal in alpha diversity, with the forearm showing the highest bacterial diversity and the scalp consistently displaying the lowest.

To unravel the paradox observed above, we propose that this discrepancy can be attributed to the combined effects of inherent limitations in traditional culture methods and skin microbial ecology. First, the inherent selectivity of traditional culture methods favors fast-growing microorganisms under artificial conditions while often suppressing slow-growing or fastidious taxa, leading to an underestimation of true community diversity^2,12^. Second, the distinct microenvironments of each skin site are crucial in shaping the observed patterns. Human skin is primarily divided into four distinct microenvironments (sebaceous, moist, dry, and specialized niches), with regions of similar microenvironments typically harboring analogous microbial community structures^2,32,33^. The forearm, as a dry site with low resident biomass, likely hosts a diversity of transient microbes from the environment. These taxa are generally poorly adapted to standard culture conditions, leading to low isolate yields; however, they are readily detected by sequencing, resulting in high apparent diversity^12,16,32^. Conversely, the scalp’s sebum-rich, moist environment provides an ideal niche for resident colonizers like *Staphylococcus* and *Cutibacterium*. These robust, well-adapted species proliferate efficiently in culture, dominating the plate counts and effectively masking rarer taxa. This ecological difference explains why the scalp exhibits high culturable biomass but lower observed sequencing-based diversity, whereas the forearm shows the opposite pattern.

Therefore, from an ecological perspective, the high sequencing diversity on the forearm reflects a mixture of transient and resident taxa amplified by methodological sensitivity, whereas the scalp’s culture dominance highlights the proliferation of a few specialized, ecologically successful residents. This ecological perspective underscores the importance of integrating culture-based and sequencing-based approaches. Culture methods excel at isolating dominant, adaptable strains, while high-throughput sequencing provides a comprehensive profile of microbial diversity, including both culturable and unculturable microorganisms. Moreover, integrating sequencing with multi-omics technologies will further deepen our understanding of the mechanisms linking skin microbiota to disease development^1,34,35^.

High-throughput sequencing analyses at both the phylum and genus levels revealed that although common microbial taxa were shared across the three sampling sites, significant intergroup differences were observed in the relative abundance of certain predominant taxa. *Cutibacterium*, *Staphylococcus*, and *Lawsonella* collectively represented over 50% of the scalp microbiome, exhibiting markedly higher abundances compared to the forehead and forearm. Prior studies have also identified *Cutibacterium* and *Staphylococcus* as the predominant bacterial genera on the human scalp, with their dysbiosis being a critical factor in the pathogenesis of dandruff and seborrheic dermatitis^36–38^. *Cutibacterium* maintains the acidic scalp microenvironment through the decomposition of sebum into short-chain fatty acids, and its depletion compromises antimicrobial defense mechanisms. In contrast, *Staphylococcus* overcolonization activates TLR2/NF-κB-mediated inflammatory signaling pathways, disrupts stratum corneum integrity and induces abnormal keratinocyte differentiation. Modulating the balance of these bacteria on the scalp, particularly by enhancing *Cutibacterium* and suppressing *Staphylococcus*, may be a potential solution to lessen dandruff^39^. Building upon these findings, future research should focus on developing precise intervention strategies to address scalp microbiome imbalance. Specifically, probiotic formulations containing *Cutibacterium* strains may restore the scalp’s acidic microenvironment and competitively inhibit pathogenic *Staphylococcus* colonization. Furthermore, integrating multi-omics technologies could establish predictive models for microbial dysbiosis and guide real-time therapeutic adjustments. These strategies have the potential to transition scalp disease management from symptom-focused control toward mechanism-driven precision medicine.

The lipophilic genus *Cutibacterium* was initially classified under the genus *Propionibacterium* but was later reclassified as *Cutibacterium* due to its distinct characteristics in origin, colonization, and genomic features^40,41^. Notable species within this genus mainly include *C. acnes* (formerly *P. acnes*), *C. granulosum*, and *C. avidum*. Among these, *C. acnes* acts as a resident microbial community and a dominant colonizer of human skin, typically inhabiting sebaceous glands and hair follicles in sebum-rich regions^42^. The scalp microenvironment is distinguished by a dense distribution of sweat and sebaceous glands, which secrete substances forming a natural water-oil barrier on the skin surface, thereby promoting the growth and proliferation of *Cutibacterium*. This explains the significantly higher relative abundance of *Cutibacterium* in the scalp compared to both the forehead and forearm sites.

Our analysis revealed that although female participants exhibited higher alpha diversity in forehead and scalp sites compared to males, along with lower relative abundances of *Staphylococcus* and *Cutibacterium* in the forehead, these differences did not reach statistical significance. The androgen-driven enhancement of sebaceous gland activity explains the significantly higher relative abundance of lipophilic taxa such as *Cutibacterium* in male skin. Conversely, the greater alpha diversity observed in female skin may be attributed to gender-specific skincare practices and cutaneous pH gradients^43^. In relation to the dynamics of age-related skin microbiota, previous studies have shown that the diversity and compositional characteristics of human skin microbiota are significantly associated with age-related changes. Immediately following birth, infant skin is rapidly colonized by microbial communities dominated by *Staphylococcus* and *Streptococcus*, exhibiting high homology with maternal vaginal and cutaneous microbiota^44,45^. Pubertal hormonal surges drive the enrichment of lipophilic microorganisms, such as *C. acnes*, alongside marked increases in microbial diversity^43^. By adulthood, the skin microbiota stabilizes into a homeostatic equilibrium and displays pronounced body site specificity^43,46,47^. These findings on the characteristics of skin microbiota in adult populations align with our observations, in which significant variations in bacterial community diversity across different body sites were detected, while no notable changes were observed among the 20s, 30s, and 40s age groups. Elderly populations exhibit increased alpha diversity, decreased *Cutibacterium* abundance, enhanced colonization by opportunistic pathogens such as *S. aureus*, and pH-dependent expansion of Gram-negative bacteria like *Acinetobacter*. These changes are collectively driven by compromised epidermal barrier function, reduced sebum production, and age-related immune dysfunction^46–51^. Overall, these results suggested that multiple host factors, such as age, gender, environmental exposure, living area, and lifestyle, have minimal influence on microbial distribution variability across human skin. Conversely, the skin microenvironment type was identified as the most important factor in shaping the community structure of the human skin microbiota^43,47,48,51^.

Many studies have characterized the microbiomes of Western individuals, while research specifically focusing on the skin microbiomes of Chinese populations remains limited. Existing studies have primarily documented skin microbiomes among residents of several Chinese cities, providing evidence that Chinese individuals exhibit distinct skin microbiomes compared to other populations, despite sharing common microbiome trends. Ying et al. conducted comparative analyses of bacterial community structures between urban and rural populations in Shanghai, revealing distinct microbial signatures between these demographic groups^43^. A Hong Kong-based study found that *Cutibacterium* (formerly *Propionibacterium*), *Corynebacterium*, *Staphylococcus*, and *Enhydrobacter* were the predominant bacterial genera in local residents’ skin microbiomes^52^. Comparative research by Kim et al. on the facial cheek microbiomes of Xi’an women across two age groups revealed that younger participants exhibited higher microbial diversity than the older group^51^. This finding was inconsistent with observations in Eastern European, Japanese, and Korean females with similar age ranges, where the skin microbiome in older groups demonstrated higher diversity and species richness than that in younger groups^48–50^. A recent investigation by Lei et al. analyzed the microbiomes of Chinese individuals across four regions (Shanghai, Chifeng, Kunming, Urumqi), revealing significant geographical variations. *Cutibacterium* dominated palmar samples from Shanghai and Kunming, while *Psychrobacter* and *Psychrobacillus* prevailed in Chifeng and Urumqi, respectively. *Streptococcus* and *Staphylococcus* consistently emerged as dominant taxa in oral and nasal cavities across all regions^53^. These findings underscore the profound impact of geographical factors on skin microbiome composition.

Our Guangzhou-based study identified distinct microbial patterns compared to previous findings from other cities. *Staphylococcus* and *Cutibacterium* predominated in both the forehead and scalp, while *Paucibacter*, unclassified Bacteria, and *Streptococcus* were more prevalent in the forearm. These inter-regional differences are likely attributable to the synergistic effects of climatic conditions, residential patterns, and lifestyle factors^49^. In summary, this study of healthy adults in Guangzhou enriches the skin microbiome database for Chinese populations, facilitating cross-regional and inter-ethnic comparisons. A comprehensive characterization of geographical variations in skin microbial composition and function will promote personalized microbiome research across diverse racial, ethnic, and cultural populations, enabling critical evaluation of the universality of current microbiome paradigms. Furthermore, these findings provide valuable insights into the mechanistic links between human skin microbiomes and dermatological pathologies.

## Conclusions

This study integrated traditional culture-based methods with high-throughput sequencing analyses to reveal distinct spatial patterning of the human skin bacterial community across the forehead, forearm, and scalp. Among 675 cultivated bacterial strains, *Staphylococcus* and *Cutibacterium* were identified as core genera shared across all sampling sites, with *S. capitis* showing marked enrichment on the scalp. High-throughput sequencing results demonstrated similar bacterial community structures between forehead and forearm sites, while the scalp microbiota exhibited significant differentiation, characterized by substantially lower alpha diversity and markedly higher relative abundance of *Cutibacterium*, *Staphylococcus*, and *Lawsonella*. Future studies should investigate how microenvironmental conditions shape microbial selection and functional changes, particularly the competitive interactions between *Cutibacterium* and *Staphylococcus* that contribute to scalp disorders. The integration of multi-omics methods will facilitate the creation of predictive models for microbial imbalance and accelerate the development of precise treatments for seborrheic dermatitis and acne.

## Supplementary Information

Below is the link to the electronic supplementary material.

Supplementary Material 1

## Data Availability

All DNA sequences of bacterial strains generated in this study have been deposited in GenBank under accession numbers PV224589 to PV225263. The raw high-throughput amplicon sequencing data have been deposited in the NCBI Sequence Read Archive under the accession number PRJNA1252278. All other relevant data are provided within the manuscript and supplementary information files.
