# Supplementary material for: A comparative analysis of skin bacteria in healthy individuals through traditional cultivation and high-throughput sequencing techniques
Source: Sci Rep. 2026 Apr 30;16:20163. doi: 10.1038/s41598-026-51175-w (PMC13324041; doi:10.1038/s41598-026-51175-w)
Supplement: Supplementary file 1 — Supplementary Material 1 [file 41598_2026_51175_MOESM1_ESM.pdf]

Supplementary information

Table S1. Gender and age distribution of 35 healthy volunteers across different sampling sites

Sampling site	Female			Male			Total
	20s	30s	40s	20s	30s	40s	
Forehead	10	4	4	4	7	6	35
Forearm	10	4	4	4	7	6	35
Scalp	10	4	4	4	7	6	35

Table S2. Time schedules for sample collection, strain isolation and identification, DNA extraction and sequencing

Sampling date and individual	Strain isolation, culturing, and identification	DNA extraction and sequencing
June 3, 2024 (15 individuals)	June 4 - July 20, 2024	June 8 - July 20, 2024
June 17, 2024 (10 individuals)	June 18 - July 25, 2024	June 22 - July 30, 2024
July 8, 2024 (10 individuals)	July 9 - August 15, 2024	July 12 - August 20, 2024
